# Supplementary material for: Association between visfatin and periodontitis: a systematic review and meta-analysis
Source: PeerJ. 2024 Mar 28;12:e17187. doi: 10.7717/peerj.17187 (PMC10981885; doi:10.7717/peerj.17187)
Supplement: Supplemental Information 1 [file peerj-12-17187-s001.docx]

PubMed: "periodontitis"[Title/Abstract] AND "visfatin"[Title/Abstract]

EBSCO: AB (periodontitis AND visfatin)

Web of science: (TS = (periodontitis)) AND TS = (visfatin)

Wiley online Library: "periodontitis"[Abstract] AND "visfatin"[Abstract]

ScienceDirect: "periodontitis"[Abstract] AND "visfatin"[Abstract]
