# Supplementary material for: Association between visfatin and periodontitis: a systematic review and meta-analysis
Source: PeerJ. 2024 Mar 28;12:e17187. doi: 10.7717/peerj.17187 (PMC10981885; doi:10.7717/peerj.17187)
Supplement: Supplemental Information 3 [file peerj-12-17187-s003.docx]

Table S2 Quality assessment for included studies

| **Author** | **Publication Year** | **Selection** | **Comparability** | **Exposure** | **Total** |
| --- | --- | --- | --- | --- | --- |
| Pradeep AR | 2011 | 2 | 1 | 2 | 5 |
| Pradeep AR | 2012 | 2 | 2 | 2 | 6 |
| Raghavendra NM | 2012 | 2 | 1 | 2 | 5 |
| Tabari ZA | 2014 | 2 | 2 | 2 | 6 |
| Mohamed HG | 2015 | 2 | 1 | 2 | 5 |
| Ghallab NA | 2015 | 2 | 2 | 2 | 6 |
| Abolfazli N | 2015 | 2 | 2 | 2 | 6 |
| Tabari ZA | 2015 | 2 | 2 | 2 | 6 |
| Özcan E | 2016 | 2 | 2 | 2 | 6 |
| Özcan E | 2016 | 2 | 2 | 2 | 6 |
| Özcan E | 2017 | 2 | 2 | 2 | 6 |
| Tabari ZA | 2018 | 2 | 2 | 2 | 6 |
| Bahammam MA | 2018 | 3 | 1 | 2 | 7 |
| Rezaei M | 2019 | 2 | 2 | 2 | 6 |
| Mopidevi A | 2019 | 2 | 2 | 2 | 6 |
| ÇETİNER D | 2019 | 3 | 2 | 2 | 7 |
| Saljoughi F | 2020 | 2 | 2 | 2 | 6 |
| Paul R | 2020 | 2 | 1 | 2 | 5 |
| Saseendran G | 2021 | 2 | 1 | 2 | 5 |
| Coutinho A | 2021 | 2 | 2 | 2 | 6 |
| Xu X | 2023 | 2 | 1 | 2 | 5 |
| Bengi VU | 2023 | 2 | 2 | 2 | 6 |
