# Supplementary material for: Association between visfatin and periodontitis: a systematic review and meta-analysis
Source: PeerJ. 2024 Mar 28;12:e17187. doi: 10.7717/peerj.17187 (PMC10981885; doi:10.7717/peerj.17187)
Supplement: Supplemental Information 4 [file peerj-12-17187-s004.docx]

Periodontitis is a chronic inflammatory disease caused by bacterial infections in the periodontal support tissue. Visfatin is mainly secreted by adipocytes and macrophages, and plays an important role in human immune regulation and defense. While, the relationship between visfatin and periodontitis remains unclear.

A total of 22 articles comprising 456 patients with periodontitis and 394 healthy individuals were included in the meta-analysis. Overall, visfatin levels were significantly higher in patients with periodontitis than in healthy individuals. And visfatin levels were significantly lower in the patients with periodontitis after treatment.

This is the first meta-analysis to compare visfatin levels between patients with periodontitis and healthy individuals.
